# Rapid prototyping of high-resolution large format microfluidic device through maskless image guided in-situ photopolymerization

**DOI:** 10.1038/s41467-023-40119-x

**Published:** 2023-07-27

**Authors:** Ratul Paul, Yuwen Zhao, Declan Coster, Xiaochen Qin, Khayrul Islam, Yue Wu, Yaling Liu

**Affiliations:** 1 Department of Mechanical Engineering and Mechanics, Lehigh University, Bethlehem, PA 18015 USA; 2 Department of Bioengineering, Lehigh University, Bethlehem, PA 18015 USA; 3 Department of Electrical and Computer Engineering, Lehigh University, Bethlehem, PA 18015 USA

**Keywords:** Microfluidics, Design, synthesis and processing, Fluidics, Polymers

## Abstract

Microfluidic devices have found extensive applications in mechanical, biomedical, chemical, and materials research. However, the high initial cost, low resolution, inferior feature fidelity, poor repeatability, rough surface finish, and long turn-around time of traditional prototyping methods limit their wider adoption. In this study, a strategic approach to a deterministic fabrication process based on in-situ image analysis and intermittent flow control called image-guided in-situ maskless lithography (IGIs-ML), has been proposed to overcome these challenges. By using dynamic image analysis and integrated flow control, IGIs-ML provides superior repeatability and fidelity of densely packed features across a large area and multiple devices. This general and robust approach enables the fabrication of a wide variety of microfluidic devices and resolves critical proximity effect and size limitations in rapid prototyping. The affordability and reliability of IGIs-ML make it a powerful tool for exploring the design space beyond the capabilities of traditional rapid prototyping.

## Abstract (web summary)

The utility of microfluidic devices has been limited by several drawbacks including low resolution, inferior feature fidelity, poor repeatability. Here the authors address these challenges by developing a strategic approach of image guided in-situ maskless lithography to fabricate a variety of microfluidic devices and resolve critical proximity effect and size limitations in rapid prototyping.

## Introduction

Microfluidics technologies have become a cornerstone in developing micron-sized total analytical systems (µTAS) and point of care (POC) medical devices in analytical, biomedical, and clinical applications^1–3^. Fabrication of microfluidic devices is largely tied to the use of clean room processes that is accessible only to trained personnel as soft lithography remains the most widely used fabrication method. Mostly suited to large-scale manufacturing, this approach impedes advancements in this field requiring significant design iterations, making it difficult to break even due to the staggering initial investment. Similar implications have hurdled research groups and industries across different fields that eventually led to the efforts to develop rapid prototyping methods. Correspondingly, there have been several noteworthy efforts to develop rapid prototyping methods for microfluidic device fabrication to make it more accessible and cost-efficient. ‘Rapid Prototyping’ is a collective term that typically means fabricating or manufacturing parts directly from a CAD file with fewer manual steps, trained personnel, tools, and time. However, most of them fail to bridge the balance between the minimum feature size and cost. For example, wax printing is one of the cheapest fabricating methods that can print ~50 µm features^4^ while on the other side of the spectrum, two-photon polymerization (2PP) can print sub-micron features but costs ~250,000 USD. Moreover, most of the one-step mold or direct device fabrication methods are based on mechanical micromachining^5^, 3D printing^6,7^, or laser ablation^8^ which results in inferior feature size and significant roughness both in the side wall and the substrate as well as reduced opacity^9^.

DMD-based maskless lithography shows promise in low-cost high-resolution microfluidic device fabrication with its user-defined pattern generation capability, rapid processing speed, and comparatively low initial cost^10,11^. Typically implemented in a thin layer of photoresist, this method often requires etching or deep reactive ion etching (DRIE) to further process the substrate before a microfluidic device can be fabricated which usually ranges from a few nanometers to even a millimeter in thickness. On top of that, most methods still require manual processes such as replicating, bonding, etc. In-situ maskless lithography may play a vital role in single-step rapid microfluidic device prototyping. In-situ polymerization of liquid-phase photocurable material along with user-defined DMD-based patterning and stop-flow control is used for the generation of uniform photopolymerized microparticles^12^, cell-laden microparticles^13,14^, permeable membrane^15^, microwell arrays^16^, etc. Photocurable materials like poly(ethylene glycol) diacrylate (PEGDA) are getting increasingly popular for microfluidic application in cell and tissue engineering^17,18^ and drug delivery^19,20^ due to their mechanical and chemical properties, resistance to protein absorption, and biocompatibility.

Polymerization during in-situ maskless lithography depends on the materials used in the pre-polymer solution, the local photochemical environment, and the UV intensity distribution. Regardless of cationic or free-radical UV polymerization, the reactive species diffuse radially outward from the excitation zone. Additionally, DMD-based illumination usually suffers from light non-uniformity^21,22^. When a nearby exposure zone is present, the combined effect of radical diffusion and light non-uniformity creates feature broadening, the over-curing, and sporadic connection between features^23^. Thus, reducing the distance between structures proves to be more challenging than reducing the feature sizes^24–26^. When generating cell-laden structures, this problem becomes more prominent due to increased light scattering by the cells, resulting in a lack of resolution and accuracy while printing^27,28^. Commonly known as the ‘proximity effect’, this can be attributed to polymer chain growth, light non-uniformity, scattering of light, thermal or molecular diffusion, and dose addition during subsequent illumination steps^23–25,28–38^. This has been a major concern during serial or parallel multi-spot lithography not only for in-situ polymerization but for maskless lithography in general^23^.

The most common approaches to resolve the proximity effect and generate high-fidelity features include manipulating the incident light by linear grating^21^, wobulation^22^, dose modulation^39^, or manipulating the pre-polymer solution itself by mixing with a photo-inhibitor. However, the effectiveness of linear grating is limited to thin photoresist layers, while the latter approach primarily improves vertical rather than horizontal spatial resolution in 3D printing^40^. In recent years, there have been significant efforts to tackle this issue. Negative projection lithography (NPL) has been proposed as a means to enhance the spatial resolution of PEGDA-based maskless lithography. This method can generate squares with a 25 µm side at a distance of 100 µm in a 20-50 µm thick microfluidic device^41^. However, NPL is a multi-step polymerization process requiring complex material synthesis. Flashing photopolymerization^28^ has been proposed to control photoinitiator radical diffusion and reduce the proximity effect. But reducing exposure time and increasing intensity may negatively affect the structure uniformity^42^. Thus, better control over exposure and pattern growth during maskless lithography is desired. Image analysis-based control in manufacturing is gaining popularity due to the advancement of cameras and artificial intelligence (AI) integration. Image-assisted closed-loop control has been used for laser-based additive manufacturing^43,44^ and their quality control^45^. The application of image-based control in 3D printing and additive manufacturing for layer height control, over and underfill correction, temperature and layer time control, print failure alert, standoff distance control, and process parameter control has been extensively discussed in recent reviews^46,47^.

In this study, we propose a systematic method of single-step in-situ prototyping to fabricate a wide variety of microfluidic devices. For the first time, image-based feedback is used for precise control of projected patterns, high-speed triggering of the UV light, and flow control for maskless lithography. This method can realize flexible process control regardless of the material, UV power, light uniformity, device thickness, or type of device. Our results demonstrate superior spatial pattern fidelity and repeatability, which we have showcased by fabricating a large deterministic lateral displacement (DLD) device. Moreover, we have demonstrated the generality of our approach by successfully patterning various photosensitive materials, including PEGDA 700, PEGDA 250, commercial resin, and gelatin methacryloyl (GelMA), which has good biocompatibility for cell encapsulation^14^ and patterning. We furthermore demonstrate the capability of the method by fabricating two vasculature network-like designs directly from a CAD file with minimum processing steps as well as generating uniform concave micropillars with tunable parameters for cell spheroid culturing. Fundamentally, we have established a systematic pathway with minimum dependencies on the materials, experimental setup, and skilled personnel to fabricate a wide variety of microfluidic devices with high spatial resolution compared to the current rapid-prototyping platforms.

## Results

**Fig. 1:**
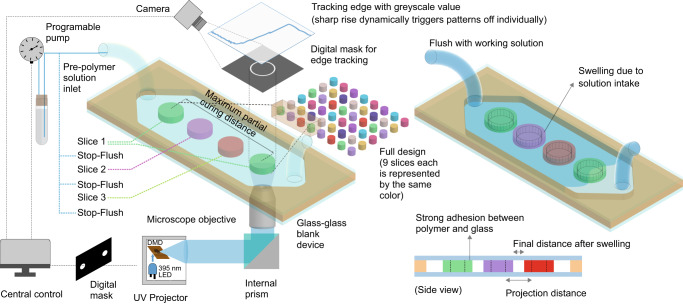
A schematic of in-situ image-guided prototyping microfluidic devices. An integrated system controls the dynamic pattern projection backed by image-based edge tracking along with the flush and stop of the pre-polymer solution. The full design is divided into multiple slices and each slice is presented with a separate color. Each slice contains patterns that are d_**critical**_ distance away from each other to avoid regional curing accumulation or over-curing. All the patterns in one slice are projected. The partially cured material is then flushed. New material is introduced before moving on to the next slice. A final flushing with the working solution is performed to enable swelling (if any) and allow the structures to reach their final size and shape.

A typical process of Image-guided In-situ Maskless Lithography (IGIs-ML) is illustrated in Fig. 1. The following sections provide an overview of the working principle of our proposed method as well as a variety of applications in the rapid prototyping of microfluidic devices. In brief, the design is first discretized into smaller patterns which are printed individually with live image-based feedback control. Slicing all the patterns in different layers and advanced flush-flow functionality improves pattern fidelity.

### Printability of features and image-guided edge tracking

**Fig. 2:**
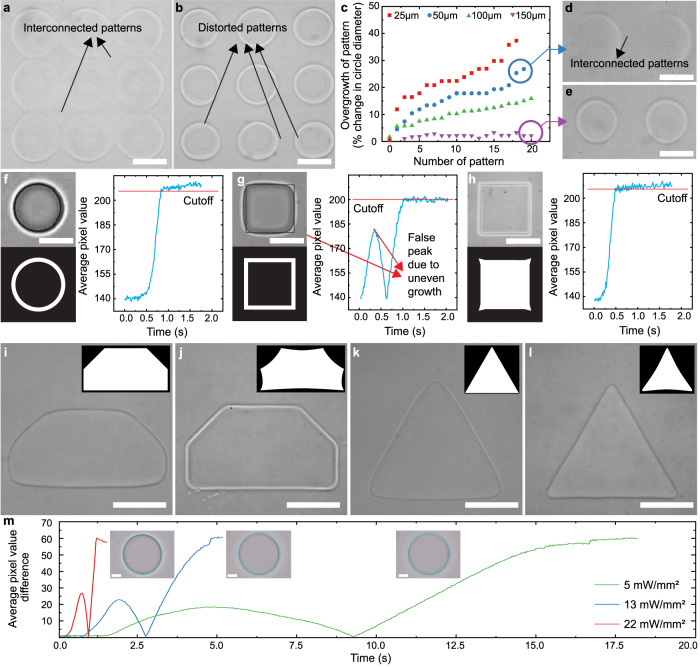
In-situ tracking and dynamic projection for maskless lithography. **a**, **b** Interconnected and distorted patterns when projecting multiple circular patterns in a ~ 50 µm thick channel at a gap of 25 µm. **c** Overgrowth in % change of circle diameter vs the number of sequential patterns (1st to 20th pattern) for different inter-pattern gaps. The plot shows how partial polymerization causes gradually increasing overgrowth with each projection when the gap is small. The gap between subsequent features not resulting in a significant increase in size during conventional projection can be considered as 150 µm for this experiment. **d**, **e** Interconnected patterns due to severe overcuring when projecting the 19th and 20th circular shapes in the sequential test at a 50 µm gap compared to the 19th and 20th circles with a 150 µm gap. **f** Left-top image shows the black outline as the desired pattern and the brightfield image of the actual pattern. Left-bottom image shows the binary mask made of 8 pixels thick annular shape centering the desired outline. This mask is multiplied with the live image to determine the mask’s greyscale value. This is tracked to determine if the edge reached the mask by setting a cutoff greyscale value. This process is shown in the average greyscale value vs time plot on the right. **g** The same process is described in (**f**) for a square pattern. This shows that for a more complicated shape, the edges can reach the mask at different times resulting in distorted patterns even with image-guided tracking. This is solved by using optical proximity correction (OPC) that results in a greyscale value without any false peak as shown in (**h**). Other shapes improved by OPC are shown in (**i**–**l**). **m** The plot of the average pixel grayscale value change in the tracking mask under various UV light powers. (Scale bar: 25 µm).

The printability of features in DMD-based maskless lithography is typically hindered by the proximity effect. In the case of closely packed patterns, this effect intensifies as a lower-degree polymerized zone at the outer portions of each polymerized structure gets re-exposed due to single or multiple exposures in close proximity^32^. This leads to feature broadening and interconnected parts^41,48^. An example of this behavior is shown in Fig. 2a, b in our experimental setup for a 50 µm thick device using 50% PEGDA 700. Figure 2a shows a 3×3 grid of circular patterns projected at once and Fig. 2b shows the same grid when each circle is printed subsequently. This demonstrates the relative contribution of spatial and temporal proximity effects in maskless lithography. In both cases, significant feature broadening is observed. As light non-uniformity and the resulting accumulating growth have a finite effective region^30^, pattern broadening inherently decreases if the distance between two features increases. We assume that beyond a certain distance, subsequent exposures have no effect on features, thus consider that as the critical distance of partial curing (d_critical_). This parameter is specific to the thickness of curing, the pre-polymer used, and the UV dose. To implement IGIs-ML, d_critical_ needs to be measured for a specific system first.

To do this, we first project four rows of 35 µm circular patterns with a fixed gap of 25 µm, 50 µm, 100 µm, and 150 µm between them, respectively. Each row contained 20 circles and was projected sequentially. When the gap between circles is small, a significant increase in subsequent circle diameter is observed. Specifically, our results showed ~36.6%, 26.6%, 20%, and 3.3% increase in the diameter from the first feature printed.

Further details of the experiment can be found in the supplementary materials (Supplementary Fig. 1) and a summary of the results is provided in Fig. 2c–e Unsurprisingly, with a larger gap distance, the pillar diameter deviation decreases. A gap of 150 µm between successive exposures results in only a 3.3% deviation in feature size.

**Fig. 3:**
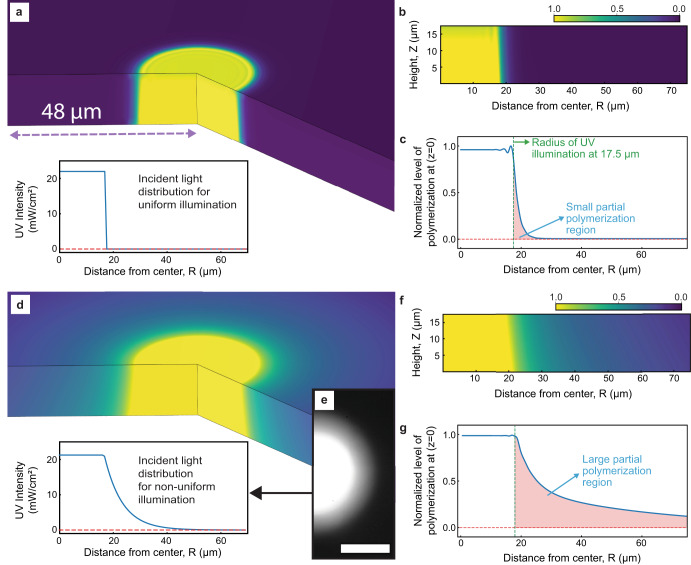
Modeling the effect of radical diffusion and light non-uniformity. **a** A cut-section and **b** side-view of normalized polymer concentration during a perfectly uniform exposure shown in the inset. **c** The normalized polymer concentration at the bottom layer for perfectly uniform illumination is plotted outward from the center. **d** A cut-section and (**f**) side-view of normalized polymer concentration during a non-uniform exposure. **e** The non-uniform illumination from the experiment is used to derive the non-uniform light profile for the simulation (Scale bar: 20 µm). This light profile is shown in (**d**) inset. **g** The normalized polymer concentration at the bottom layer for non-uniform illumination is plotted outward from the center.

We use a simplified reaction-diffusion kinetic model^42^ for free-radical polymerization to simulate the effect of the reaction kinetics and light non-uniformity in feature broadening under configurations similar to the experiment described above. The simulation results, summarized in Fig. 3, indicate that a perfectly uniform light exposure results in a small partially polymerized zone driven by the reaction kinetics until the diffused radicals are terminated by the polymerization. The illumination during the experiment, however, shows a gradual decay of intensity outward of the illumination zone instead of a sudden drop. Applying such exposure to simulation results in a much larger partial polymerization zone which extends >70 µm from the edge of the pattern. This may explain the observed feature broadening in consecutive patterns printed at distances below 150 µm. A detailed description of the simulation can be found in the methodology section. Furthermore, we conducted a simulation with the same model of two exposures at various distances using both uniform and non-uniform illumination, resulting in patterns consistent with experimental observations, as shown in Supplementary Fig. 2. The propagation of the normalized polymer concentration for both cases is shown in supplementary movie 2.

Evidently, for the specific setup, individual patterns projected at >150 µm distance show almost no proximity effect which is considered as the d_critical_ for our experiment. We propose a simple method that divides the patterns into slices where each slice only contains features that are at least a d_critical_ distance away from each other. Then it sequentially projects one slice followed by flushing the partially cured materials from the device, replacing it with a fresh pre-polymer solution, and then printing the next slice. It is possible to set the d_critical_ larger than what is suggested by the calibration experiment. This will result in more slices during the fabrication. However, each slice increase will only add the time equivalent to one ‘flushing time’ to the overall fabrication process.

To improve the repeatability of printing and develop a generalized printing quality control, we first analyzed the UV polymerization process with a high-speed camera. As anticipated, the high-speed imaging reveals an edge around the pattern being projected that can be distinguished by the difference in the greyscale value which can be easily traced. A thin 10-pixel binary mask centered at the outline of the pattern is applied to the live image to serve as a detection boundary so that the exposure will stop immediately once the cured pattern edge touches it. The greyscale values of the masks are tracked at 100 fps and each pattern is turned off independently (when multiple patterns are projected at the same time) once a pre-defined cutoff value is reached indicating the edge reaching the desired boundary location. The whole process is summarized in Fig. 2f, g for two typical patterns: a circle and a square. It is also shown in the supplementary movie 1 for 4 circular patterns where the top left pattern was given a smaller UV dose to simulate uneven exposure. The movie shows how the masks can be turned off dynamically based on edge tracking. The movie also shows an important phenomenon for patterns containing angles. Due to the uneven growth of the square, two peaks can be seen in the pixel value vs time plot which indicates the sides of the square reaching the edge at different times. This can be solved by optical proximity correction (OPC) which is a common technique in photolithography to compensate for the error due to diffraction^49,50^. The fact that the projection process is image-guided makes it convenient to apply OPC as shown in Fig. 2h–l. The standard pillars with different shape complexities are changed based on OPC to account for the rounding due to the curing process around corners. The new shapes are created using a generalization of the Fernandez-Guasti Squircle Equations which were developed to translate circles into squares. In this case, the same transformers are used to transform the angles of the shapes to corrected versions which will allow for higher accuracy near the corners.

**Fig. 4:**
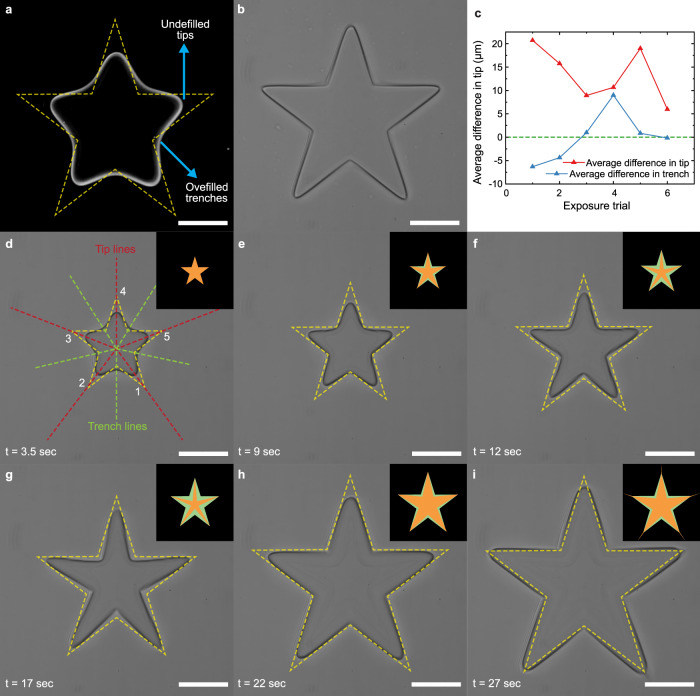
Dynamic shape correction during photocuring. **a** Edge extracted from the images during the fabrication process. Overfilled trenches and underfilled tips are observed during the initial shape projection. **b** The final shape after dynamic pattern correction projection. **c** The plot of the average deviation of tips and trenches from the desired shape during iterative shape correction. **d**–**i** A total of six projection iterations are performed with the shape being projected in orange and the standard shape in light green in the inset. **d** also illustrates the lines along which the edge of the cured pattern is being tracked (Scale bar: 100 µm).

OPC is an offline process that requires post-fabrication analysis of printed patterns. However, live image feedback and edge tracking can also be capitalized to perform dynamic in-situ pattern correction if a correlation between the current and desired shape can be established. Figure 4 shows a simplified dynamic pattern correction where a star-shaped feature with sharp corners is printed by intermittently changing the pattern. The patterns are modified based on the location of the tips and trenches of the polymerized material which are tracked by live edge tracking. A star-shaped pattern has sharper angles compared to a circle or a rectangle, thus is chosen as an example to represent a more general case of complex pattern. The method is described in the methodology section in detail and the dynamic process is shown in supplementary movie 3.

The change in the greyscale value of the mask may depend on the focus of the microscope. However, the edge can be detected regardless of the focusing condition up to a certain level. Supplementary movie 4 shows the growth of a 100 µm circular pattern in a 50 µm thick blank device using 50% PEGDA 250 with UV power of 22 mW/mm^2^, 13 mW/mm^2^, and 5 mW/mm^2^, respectively. In each case, the curing process shows a white halo followed by a black edge. Figure 2m and supplementary movie 4 show each pattern results in a distinct curve with two peaks. The first one is caused by the white halo reaching the mask while the second peak is due to the dark edge. Either peak can be utilized as a trigger to turn off the UV light.

So far, we have demonstrated that edge tracking, dynamic pattern correction, and offline OCR can be performed in PEGDA 700 and PEGDA 250 with various pattern sizes and shapes and different UV exposures. This also shows that the speed of the curing process can be easily controlled by modulating the UV power without affecting the final shape and size of the pattern. As a result, edge tracking can be performed irrespective of the processing power of the setup. It should also provide additional flexibility in implementing advanced decision-making based on the curing process.

### Improved in-situ printability

**Fig. 5:**
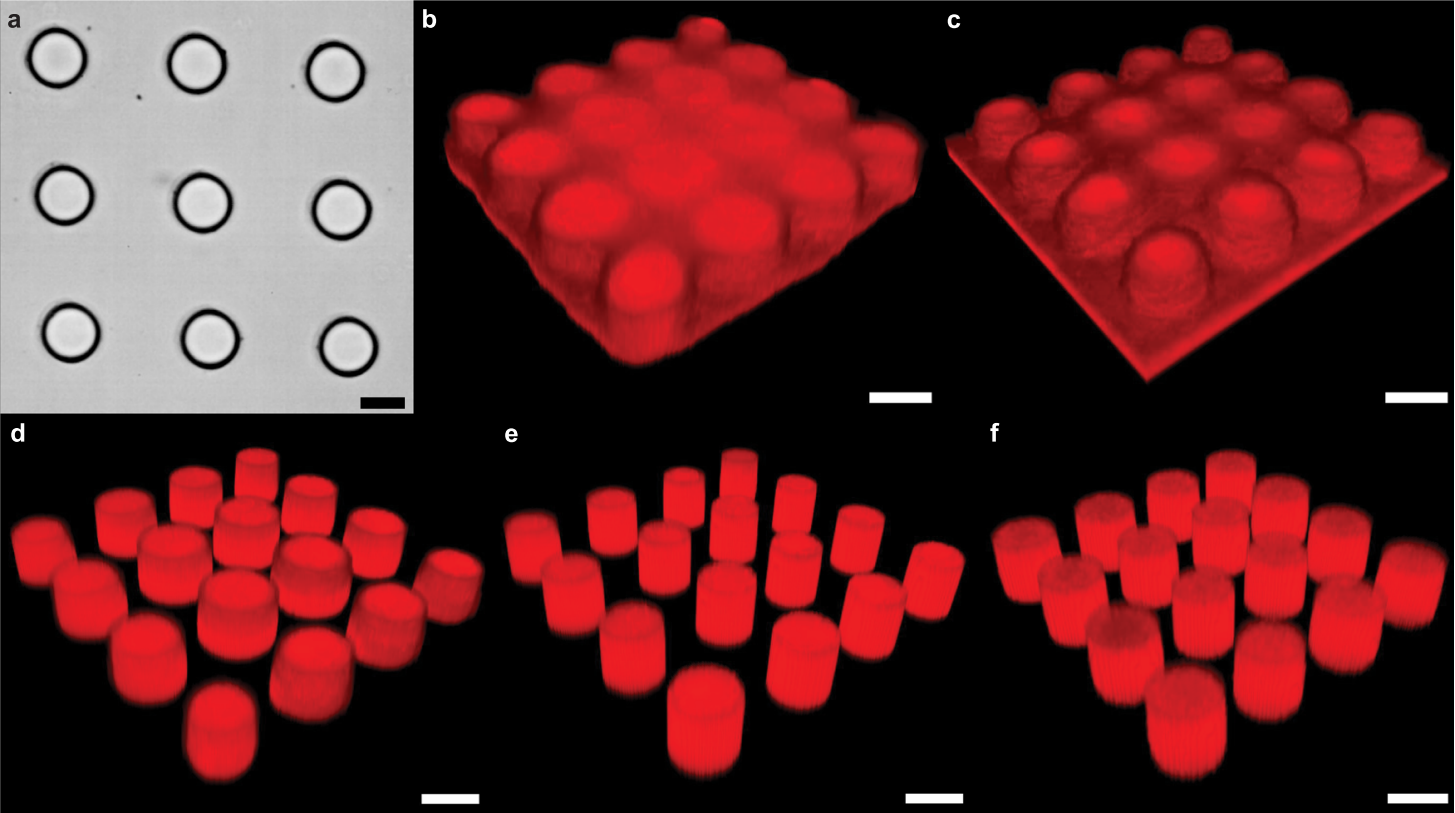
Printed micro-patterns with the proposed IGIs-ML method compared to the conventional methods. **a** Brightfield image of 25 µm pillars printed at 30 µm gap. **b**–**e** The same patterns were projected with conventional single projection, consecutive pattern projection, edge tracking without flushing, and edge tracking with flushing of the partially cured pre-polymer solution, respectively. **f** Projecting the same patterns with a  µm gap with IGIs-ML (Scale bar: 25 µm).

IGIs-ML significantly improves the printability of densely packed structures during in-situ fabrication. Figure 5a shows a brightfield image of a 3×3 grid of circular patterns projected in a 50 µm thick blank channel with 50% PEGDA 700 which demonstrates clear improvement from the patterns shown in Fig. 2a, b due to the implementation of IGIs-ML. Figure 5b–e shows the 3D reconstructed confocal images of a 4×4 array of 25 µm pillars placed at a 30 µm gap distance patterned in a ~ 30 µm thick blank device with 50% PEGDA 700 using 1 s of 22 mW/cm^2^ exposure with four different methods. (i) Single exposure of the full set of pillars, (ii) 16 successive exposures of single pillars starting from top left to bottom right, (iii) 16 successive exposures with flushing off partially cured material between exposures without edge tracking, (iv) 16 successive exposures with flushing and edge tracking. Conventional exposures (i and ii) clearly show interconnected pillars with the center of the patterns being the most over-exposed region. But more importantly, although flush flow removes the lower-degree polymerized pre-polymer before each exposure, the pillars form more abruptly when surrounded by cured regions as seen for case (iii) in Fig. 5d. As a result, the same exposure dose generates slightly enlarged features. Thus, image-guided triggering is essential for maintaining repeatability. Figure 5f shows similar pillars printed by IGIs-ML with a 10 µm gap.

### Prototyping of a large array of structures for DLD application

To justify the effectiveness of IGIs-ML in the rapid prototyping of microfluidic devices we first demonstrate the fabrication of a deterministic lateral displacement (DLD) device that is widely used for size-based separation of particles^50,51^. Besides being a large device ranging up to a few centimeters in length, this application relies upon subtle interactions between particles and micro-posts even a small size difference will dramatically affect device performance, thus requiring precise feature size and placement.

**Fig. 6:**
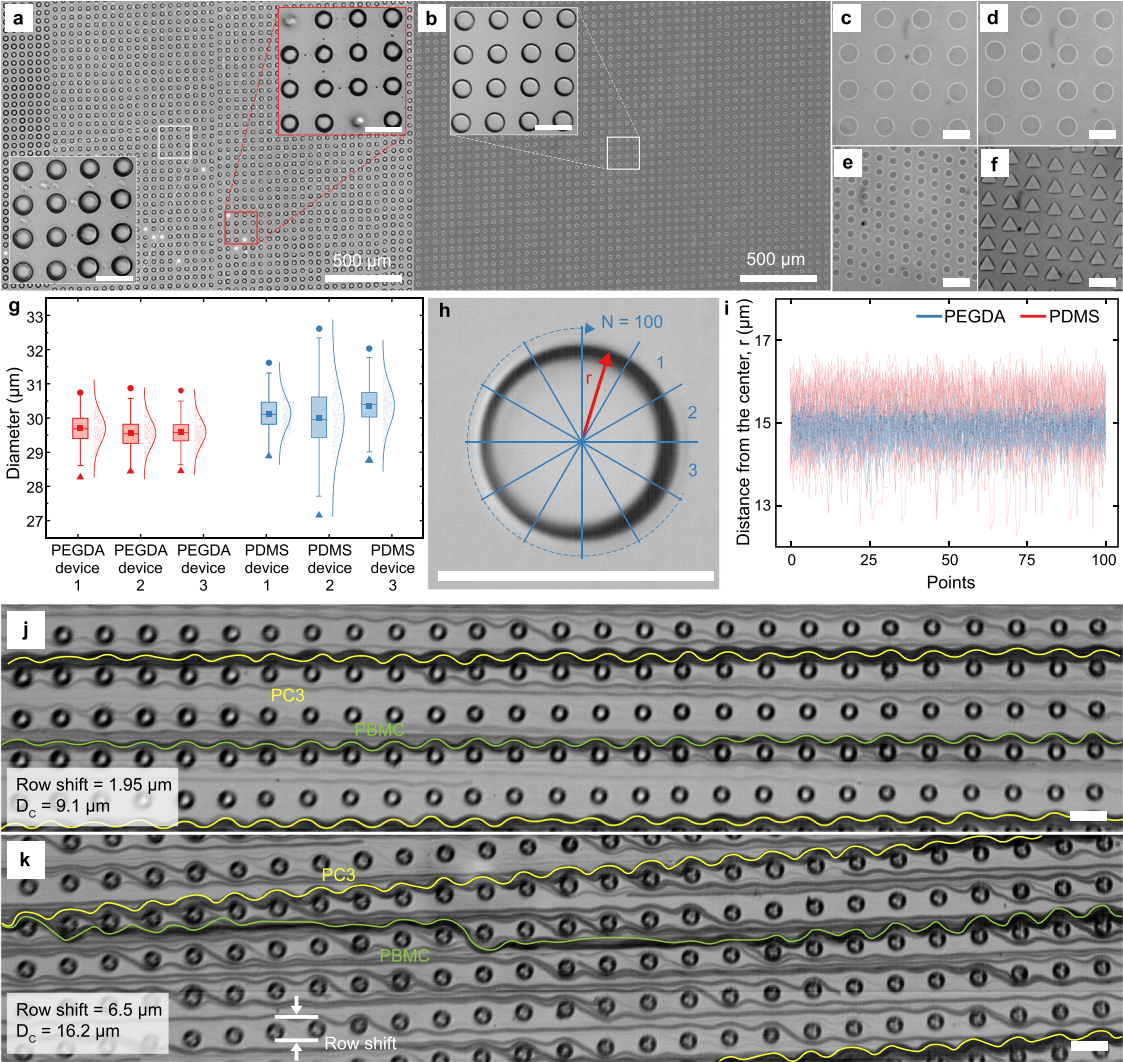
Repeatability of the proposed method for large-scale fabrication. **a** PDMS device fabricated with conventional photolithography using a 64k DPI chrome photomask shows a portion of a typical DLD device with a 29.6 µm pillar size, 26.4 µm gap, and 2 µm row displacement. **b** the same DLD configuration printed with the proposed method with 50% PEGDA 700. **c**–**f** Different configurations of densely packed pillars with the smallest feature of 8 µm and the minimum gap of 7 µm. **g** Distribution of the pillar size for 3 different DLD devices with the aforementioned parameters showing the narrower distribution of median pillar diameter of PEGDA device when compared to PDMS devices made with conventional photolithography. The box-plots presents the diameters of 500 printed circle in µm for PEGDA 1 to 3 and PDMS 1 to 3 with minima (28.27, 28.44, 28.45, 28.88, 27.15, 28.77), maxima (30.74, 30.87, 30.8, 31.61, 32.61, 32.03), median (29.68, 29.54, 29.58,30.1, 29.96, 30.4), 1^st^ quartile (29.34, 29.26, 29.82, 29.42, 30.03), 3rd quartile (29.99, 29.83, 29.8, 30.46, 30.61, 30.75) and lower whisker (28.51, 28.42, 28.62, 28.86, 27.64, 28.95), upper whisker (30.88, 30.65, 30.51, 31.42, 32.97, 31.82) and 95th percentile (30.42, 30.24, 30.3, 30.89, 31.44, 31.26) respectively. (**h**, **i**) The smoothness of the outer profile of the patterns was calculated and compared with PDMS patterns by plotting the radius at 100 different positions for 50 pillars. **j**, **k** Trajectories of two types of cells, PC3 and PBMCs in two different designs of PEGDA device with critical diameters of 9.1 µm and 16.2 µm, respectively. (Scale bar: 50 µm unless otherwise mentioned in the figure).

Due to the deterministic control for every single pattern being projected, IGIs-ML shows substantial uniformity in pattern size and location. Figure 6a, b shows a comparison of a DLD device with a 29.6 µm pillar diameter, 26.4 µm gap, and 2 µm row displacement fabricated with standard soft lithography using a 64k DPI chrome mask (a) and through IGIs-ML (b). Figure 6a shows some common issues associated with pattern replication on PDMS from SU8 master fabricated using photolithography. If the SU8 coating is not completely uniform, a mismatch of pattern size can be observed due to uneven exposure. In addition, if the small trenches in the SU8 master are not completely developed, there will be a mismatch in pillar height resulting in delamination, as shown in Fig. 6a left inset. Demolding can result in missing pillars which are shown in the right inset. These anomalies become significant for sensitive applications such as DLD.

IGIs-ML provides superior repeatability and uniformity as illustrated in Fig. 6b. Figure 6c, d shows 35 µm pillars fabricated at a 30 µm gap with target row shifts of 1.3, and 2.6 µm. After fabrication, the measured row shift is 1.51, and 2.87 µm respectively, resulting in an accuracy of ~200 nm regardless of the gap and shift size. The fabrication time directly correlates with the size and design complexity. For example, a 5 cm long and 5 mm wide DMD device mentioned above will contain ~60,000 pillars which usually takes 2 h to make. Smaller domains can be fabricated with proportionally decreased fabrication time. A 2 mm × 2 mm pattern area can be fabricated in <10 min. Standard soft lithography could generate more devices at the same time once the design is finalized. However, during design modification or testing, it takes several days to weeks of waiting time and several working hours to generate a mold for each design depending on the type of mask, mask vendors, clean-room facility, and the person who is making it. In comparison, the only manual steps for IGIs-ML are CAD drawing and making easy-to-make blank devices, and the rest is done automatically, thus saving many days of waiting and hours of working time. A typical DLD fabrication process is partially shown in supplementary movie 6. Figure 6e, f shows 20 µm diameter pillars and triangles with 25 µm side lengths printed with 7 and 10 µm gaps seamlessly. The proposed method also shows superior pattern uniformity among multiple fabricated devices compared to PDMS devices fabricated with a standard lithography-molding approach, as shown in Fig. 6g.

Another important parameter in microfabrication is the roughness of the patterns. The roughness of the printed pillars has been analyzed from over 50 pillars by measuring the distance of the edge of the pillars from the center at 3.6° intervals following the image processing algorithm explained in another study^52,53^ and is shown in Fig. 6h, i. Pillars generated with the proposed method have much better smoothness over PDMS patterns made from a SU8 mold generated by standard photolithography using a 64k DPI chrome mask. The Root Mean Square (RMS) value of the deviation of the distances from the center provides a quantitative measure of the roughness^53,54^. The RMS of the pillar diameter for PEGDA and PDMS devices are 0.79 µm and 1.99 µm, respectively, meaning PEGDA has a much smoother side surface profile than PDMS devices.

We test the devices to check the displacement for cells of different sizes during a DLD experiment. When the critical diameter (D_c_) of the pillar array is 9.1 µm, PC3 cells, and PBMCs mainly showed bumping, as shown in Fig. 6j. PBMC sizes range from 8–10 µm, and a small number of PBMCs appeared to be zigzagging. With a D_c_ of 16.2 µm, the D_c_ was significantly larger than the size of the PBMCs but smaller than most of the PC3 cells that range from 16-20 µm. So, the trajectory shows that the PC3 maintained bumping while PBMCs start to zigzag as seen in Fig. 6k.

### CAD-to-device one-step microfluidic device prototyping

The previous section shows the accuracy of IGIs-ML in fabricating discrete patterns over a large region. In a more general case, a microfluidic device could consist of large continuous, or interconnected channels. So, here we demonstrate the capability of IGIs-ML to fabricate several typical microfluidic devices in glass-glass and glass-PDMS blank channels.

PEGDA 250 is selected for this application as it has significantly less post-cure swelling compared to PEGDA 700^55,56^. For repetitive non-connected patterns, the post-curing swelling due to water intake does not affect the structures. In the case of interconnected patterns, the swelling of patterns will push themselves away from each other resulting in distorted and blocked microfluidic channels. PEGDA 250 patterns do not swell and retain the desired shape after washing and while using the device. While the glass-glass device is suitable for several microfluidic applications, the use of PDMS devices is more standard in cell culture-related applications due to gas permeability. That is why we have demonstrated that the proposed method can be used to generate microfluidic channels inside glass-PDMS devices without any modification. It should be noted that due to oxygen inhibition, there is an uncured layer at the interface of the PEGDA-PDMS. For a 50-100 µm thick chamber, such layer thickness is usually in the range of 2–3 µm^56,57^. In the proposed method, the PEGDA is anchored at the bottom of the glass interface. Thus, the gap in the PDMS interface does not significantly affect the downstream cell seeding, culture media flow, and cell growth.

**Fig. 7:**
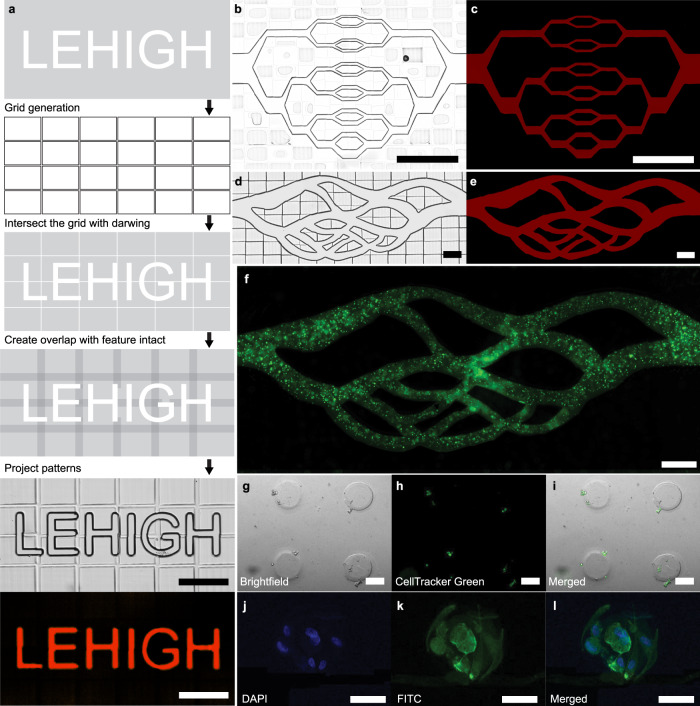
Automated in-situ micro-vessel and cell-laden hydrogel fabrication. **a** Pre-processing and process steps for fabricating microfluidic channels in situ directly from the CAD drawing. **b**–**e** Two types of vasculature network designs printed using IGIs-ML inside a glass-glass (**b**, **c**) and glass-PDMS (**d**, **e**) blank channel. Both brightfield and fluorescent images are shown that indicates no leak. **f** HUVECs laden inside of the PEGDA vessel network scaffold stained by the Calcein AM. **g**–**i** Printed micro posts with fibroblasts encapsulated in GelMA, and (**j**–**l**) fibroblast cluster growth after being embedded in GelMA for 5 days. (Scale bar: 200 µm (**a**–**f**) and 50 µm (**g**–**l**)).

As the first example, vasculature networks with well-defined hierarchical channels and natural geometry are fabricated directly from a CAD file. The full drawing is divided into several individual patterns during the pre-processing step. These patterns are then projected following the slicing, flush-flow, and image-guided approach described in the previous sections. This results in seamless stitching of the design without any complex steps and generates small channels without blocking. The pre-processing steps are illustrated in Fig. 7a and explained in detail in the methods section for generating the text LEHIGH inside a 100 µm thick glass-PDMS blank channel with 95% PEGDA 250. A typical fabrication process from a CAD file is partially shown in supplementary movie 7. Two vasculature network designs are printed, washed, and filled with 5% Rhodamine B in PBS before imaging Fig. 7b–e. The fluorescent images confirm that there are no openings or leaks between the blocks. We were able to fabricate ~5 µm wide and 30 µm tall channels conveniently.

The system shows a strong capability of generating high-resolution and versatile vessel scaffolds. With intricate fractals to mimic a natural vascularized structure, and evaluate the cell patterning ability of the scaffolds, we seeded Human umbilical vein endothelial cells (HUVECs) into the vessel network fabricated inside a glass-PDMS blank device by mixing cells with 5 mg/ml fibrin gel. Figure 7f shows the fluorescence image of HUVECs. Here, cells were stained in green with the Calcein AM and distributed within a printed vasculature pattern from a scanned image of real vasculature. Instead of encapsulating cells directly into PEGDA or attaching them to a glass surface to form a monolayer, cells were mixed with ECM (Fibrin gel) to build a cell-laden structure. Results of cell seeding and culture experiments in printed scaffolds demonstrate the patterning capability of IGIs-ML for live cell applications.

As the second example, we demonstrate the patterning of a biocompatible hydrogel. GelMA was used as a bio-ink because of its intrinsic cell adhesion moiety that promotes cell spreading and function^58,59^. Therefore, we encapsulated the normal Human Lung Fibroblasts (nHLFs) within the cured GelMA to verify the feasibility of the platform for 3D live cell patterning. After 5 days of culture, nHLF cell clusters showed proliferation and formed fibrous structures, as shown in Fig. 7j–l.

### Greyscale lithography for a large array of uniform 2.5D structures

Recently unorthodox microfluidic devices with complicated 3D structures are becoming popular. One example is microfluidic chips integrated with microwell arrays for cell trapping^59,60^. The proposed method can be combined with greyscale maskless lithography to generate semi-3D microstructures. Here we demonstrate an easy and convenient way to fabricate uniform concave micropillars with controllable size and shape made from commercial resin which is then used as a mold to make microwells. Concave micropillar arrays of different sizes and shapes have been printed inside a glass-PDMS channel. The blank channel has a significantly larger thickness than the desired height of the microwells. A commercial resin (Biocompatible Resin for Surgical Guide, Power Resins) is used for this application as they have much higher strength compared to PEGDA and thus facilitates the molding process without damage. Due to the greyscale pattern, a definitive edge is not observed during the fabrication. Thus, only slicing and flush-flow are used for this process to avoid excessive polymerization during fabrication. The current microwell fabrication process is largely dependent on tedious multistep lithography or mechanical machining. Commercial microwells are expensive and lack flexibility in design. CO_2_ laser micro-milling is perhaps the most commonly rapid fabrication method of uniform concave microwell so far^42,60^.

**Fig. 8:**
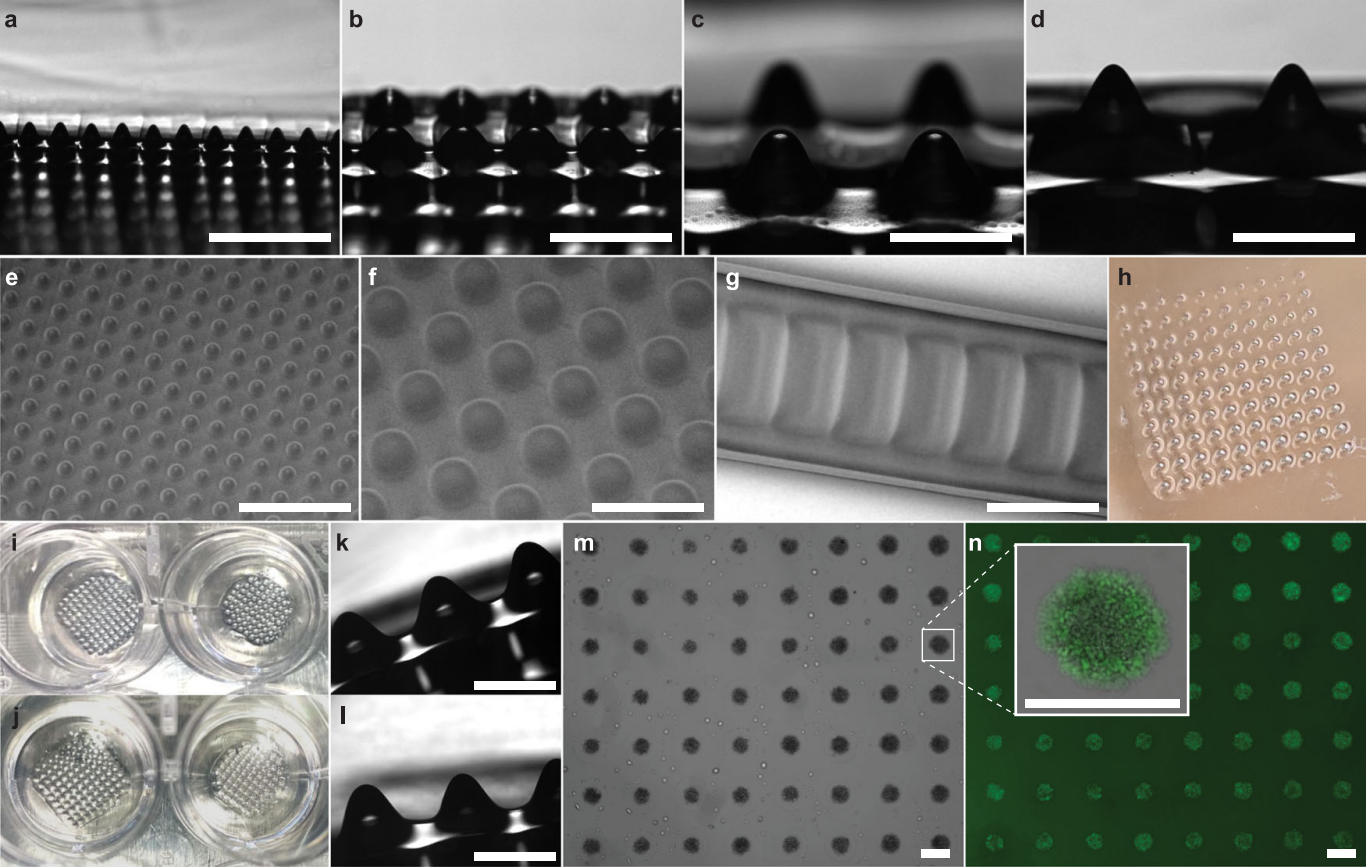
Patterning large area of uniform 3D structure using greyscale lithography by the flush flow of partially cured resin. **a**–**d** Concave pillars of different sizes fabricated with commercial resin by IGIs-ML. SEM images of the concave micropillars (**e**, **f**) and wavy pattern (**g**). Microwell printed on polystyrene slide (**h**) and PEG gel (**i**, **j**) from the master mold. **i**, **j** Before and after the molding process in PEG gel. **k**, **l** Before and after images of the master mold during the molding process which show no visible damage to the master. **m**, **n** Uniform 3D HCT-116 cell spheroid grown inside the fabricated microwells. (Scale bar: 500 µm).

Figure 8a–d shows the side view of an array of micropillars with various profiles fabricated through the procedure described in the previous section. Figure 8e–g shows SEM images of the microwell arrays and a wavy pattern demonstrates the uniformity of the fabricated features. As a result, this can easily be used to replicate microwells in a polystyrene slide which is shown in Fig. 8h without any damage to the mold. We also demonstrate the fabrication of microwells in PEG-based gel that are shown in Fig. 8i, j. Figure 8k, l shows images of the pillars before and after molding that show no visible damage to the master mold. Figure 8m, n shows brightfield and Live-Dead staining of 3D cell spheroid (HCT-116) cultured inside the PEG gel-based microwell. This shows the formation of uniformly sized cell spheroids in a massive array of microwells.

## Discussion

This work presents a maskless lithography-based rapid prototyping methodology to fabricate microfluidic devices in situ with image-assisted photopolymerization and advanced flow control called IGIs-ML. Based on the examples shown, we can claim that the proposed method can streamline the fabrication process of a wide range of microfluidic devices regardless of the materials or setup used. The proximity effect and feature broadening are not limited to in-situ polymerization. It is a well-known phenomenon in lithography, laser-writing, two-photon polymerization, and 3D printing. We have not yet examined if UV exposure in photoresists exhibits any distinct edge in the image, but naturally, they are incompatible with the flush-flow approach. However, IGIs-ML is better suited for single-step UV polymerizable materials and can be implemented in existing in-situ polymerization platforms. Additionally, it can be integrated into SLA or the VAT polymerization process, provided that proper imaging and pre-polymer flow control can be achieved. By eliminating the need for precise calibration of materials and environment, image-based pattern control could simplify the exploration of new photocurable and bio-compatible hydrogels. Furthermore, image-tracking-based fabrication has great potential for microfluidic device fabrication and integrating data-driven and intelligent manufacturing, which is becoming increasingly popular.

## Methods

### Preparation of the pre-polymer solution

Poly (ethylene glycol) diacrylates (PEGDAs) of Mw 250 and 700, and a photoinitiator, 2-Hydroxy-2-methylpropiophenone (Irgacure 1173) were purchased from Sigma-Aldrich. The Gelatin methacryloyl (GelMA) and Lithium phenyl-2,4,6-trimethyl-benzoyl phosphinate (LAP) were purchased from Cellink and Allevi, respectively. PBS and DPBS were purchased from Fisher Scientific. Irgacure 1173 is soluble in pure PEGDA, whereas PEGDA 250 prepolymer solution is prepared with 1% v/v Irgacure 1173 added to the PEGDA 250 solution. For the PEGDA 700 prepolymer solution, different volumes of PEGDA 700 and PBS were mixed in a beaker to protect from the light. The 1% v/v Irgacure 1173 was added into the solution and stirred by magnetic rode until fully dissolved. The GelMA prepolymer solution consisted of 10% w/v GelMA and 1% LAP in DPBS.

### Preparation of the blank device

A blank device is required to facilitate IGIs-ML which defines the outer boundary of the device and contains all the internal structures. The blank device is generated using materials that are readily available in the lab. Tapes of different thicknesses are used as spacers while commercial resin is used as the binding material for glass-glass devices. As the small features are fabricated using maskless lithography, there is no heavy requirement to fabricate the mask for the blank channel. A plastic mask or a cut-out sticker (Silhouette HD) is used as the mask. Inlet and outlet holes are made with a water jet on the top glass slide. Then the two glass slides are separated by a spacer and resin. The mask is placed on top of the slide aligning the inlet and outlet before making the exposure using a standard UV flashlight. The residual uncured resin is then washed off using DI water and isopropyl alcohol (IPA) and then dried at 55 °C for an hour. Pieces of PDMS with holes punched on them are plasma treated and bonded over the inlet and outlet holes to help fasten the inlet and outlet tubes as shown in Supplementary Fig. 3.

The glass-PDMS blank device is fabricated by using large PDMS channels. Holes are created in the PDMS using a biopsy punch. The PDMS part is then cleaned and dried in the oven at 55 °C for an hour before using oxygen plasma treatment to seal it with the glass slide.

### Experimental setup and process design

**Fig. 9:**
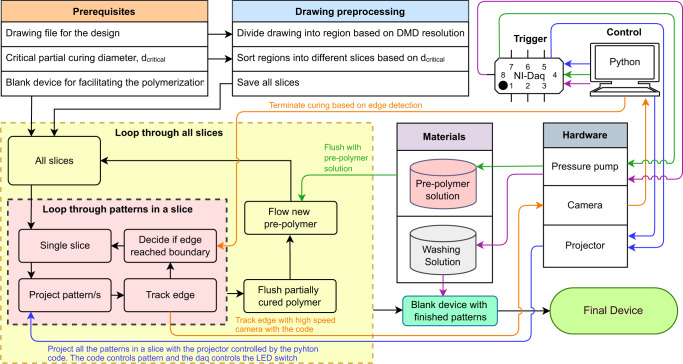
Flow diagram of the proposed IGIs-ML method. The figure shows a detailed process flow to implement the IGIs-ML.

First, the prepolymer solution is pumped into the blank device. The dynamic projection is done with a standard UV maskless lithography setup. A UV projector (In-Vision Phoenix) equipped with a 395 nm UV LED capable of generating maximum power of 22 mW/cm^2^ is placed at the field aperture of an inverted microscope (Nikon Eclipse TE2000S). The projector is equipped with a 1920×1080-pixel DMD (Texas Instrument DLP 670 S) which generates patterns by redirecting the incident light by mechanically rotating ±12°. The setup is shown in Supplementary Fig. 2 and supplementary movie 5. The pattern is then directed towards the blank channel by the internal optics of the microscope. First, the internal features of the device are divided into many unit area projections. Each unit projection has a spatial size limit that depends on the objective used. Patterns smaller than the spatial limit are kept as is while larger regions are divided into smaller ones. Once all the regions are created, we first calculate d_**critical**_ and any regions that are d_**critical**_ distance away from each other will be projected with image-guided projection described in the results section using a precision X-Y microscope stage (Zaber X-ASR) to move the blank device. The image is captured with a high-speed camera (Ximea XiB CB 120-CM using XIMEA CamTool V4.21.29) with on-the-fly processing using XIMEA API for Python 3.8 with Spyder 5.4.3. The partially cured material inside is flushed away and replaced with fresh ones before projecting the next set of regions eventually completing the projection of the whole design after several iterations. The process has been tested in two types of blank devices, (i) glass-PDMS and (ii) glass-glass with different types of pre-polymer solutions such as PEGDA 700, PEGDA 250, GelMA, and commercial biocompatible resin. After the initial fabrication, the device is washed with the working solution where the washing time may vary depending on the size and complexity of the design. Some crosslinked polymers such as PEGDA 700 may show swelling as the internal mesh absorbs water molecules whereas others may show less (PEGDA 250) or no (commercial resin) swelling. A detailed flow of the process for IGIs-ML is shown in Fig. 9. Offline image processing was done using ImageJ 1.53n.

### Modeling of the polymerization process

A simplified reaction-diffusion kinetic model is used to simulate the pattern polymerization process in our experiment, based on initiation, polymerization, and termination of free radical polymerization. Proposed by Park et al.^42,49^, the model assumes that light converts the photoinitiator into an activated species which then converts a monomer into a free radical monomer. The radical monomer reacts with a monomer to form a polymer. Termination occurs when two radicals react or when a radical reacts with a terminator, resulting in the consumption of the radical and the formation of a polymer. The model neglects the distribution of polymer chain lengths and the fact that polymers and radicals are attached. It also assumes that cross-linking is negligible and that the diffusion constant of a polymer is zero. COMSOL 5.6 is used to run the simulation.

As the experiment is carried out inside a glass-glass device, the terminator concentration is considered to be zero. A piecewise equation has been used to implement the non-uniform illumination while keeping the maximum exposure power at 22 mW/cm^2^ according to the specifications of our UV LED. The exposure time *T*, device height *H*, and pattern radius *R* have been kept constant at 1 sec, 20 µm, and 17.5 µm respectively. The concentration of polymer after 1 s is normalized by considering the highest value of the spatial concentration to be 1 and is denoted by the normalized level of polymerization. A polymerization level of 1 is considered fully cured while a level below 1 is considered partially cured. Removal of partially cured PEGDA has been demonstrated in generating multiplanar nanowrinkled surface^61^. From our experiment, a significant improvement in printability is observed due to flush-flow. Consistent with previous report^42,49^, the maximum polymer concentration is seen as a ring on the edge of the pattern when a high-intensity UV with a short exposure time is used.

### Image-based dynamic pattern distortion correction

To demonstrate dynamic pattern distortion correction during fabrication, we generated a ‘Star’ shaped structure with multiple sharp angles which is usually hard to maintain precise shape during photopolymerization in thick photocurable materials. A 50 µm thick device has been used with 95% PEGDA 250 for this experiment. The sharp angles form both tips and trenches. As a result, while sharp tips can be realized by increasing the UV power and exposure time, they will lead to more rounded trenches. This opposing effect makes it an appropriate example to demonstrate a simple feedback loop for dynamic pattern generation.

In brief, a star-shaped structure with minimal deviation in the sharp angles is fabricated by dynamically changing the projected pattern based on the deviation of the intermediate structure from the desired shape. A lower UV dose is used for this experiment which makes the curing process purposely slower without significantly changing the outcome as shown in Fig. 2m. The initial projections are smaller than the final desired size. Initially, a standard shape is projected, and the cured structure edge is analyzed to determine the deviation of the corners from desired locations. The outer and inner points are considered as tips and trenches here. By drawing a line from the center to all the tips and trench points, the location of the actual edge is tracked. If the edge of the tips or trenches is leading it means overfill while a trailing edge means underfill. The trench angle is reduced or increased if the trench edge is leading or trailing, respectively. The opposite is done for the tips or outer angle. We showed 6 iterations and reached a deviation of <5 µm on all the edges. The process is shown in supplementary movie 3.

### Pre-processing of DXF file for automated fabrication

First, a grid is generated based on the maximum projection area of the projector. A grid of 100 µm smaller than the allowable DMD resolution is generated and intersected with the desired design to generate individual patterns for a sequential project. To confirm the proper stitching of the channel, each pattern is expanded by 50 µm on each side before subtracting the channel from the expanded patterns to keep the channel region unaffected. Then this modified CAD file is sent for the fabrication process. The file containing the entire internal design of the microfluidic device is automatically processed using the ‘EzDXF’ library in Python which iterates through all the individual patterns and saves them as an individual binary mask along with their center locations. In the next step, the patterns that have centers at >d_critical_ distance away from each other are projected. All the patterns thus are projected with successive flushing in between. The initial DXF file containing the drawing was created using Autodesk Autocad 2023.

### Microwell replication with resin mold

Microwell replication has been demonstrated both in hard (polystyrene) and soft (gel) materials. For patterning in polystyrene, a polystyrene slide was placed over a glass slide. The mold of concave micropillars is placed over the polystyrene slide before using another glass slide to sandwich them. This is fastened with two binder clips and placed at 55 °C for 6 h.

The hydrogel was formed via a Michael-type cross-linking reaction by mixing 4arm-PEG-SH (10 kDa) and 8arm-PEG-VS (10 kDa) at room temperature. The PEG macromers were mixed and diluted to 5% (w/v) for curing with triethanolamine buffer (0.3 M (pH 8); Sigma-Aldrich). The fabricated microarray mold was pressed into the hydrogel after 5 min when the gelation occurred at room temperature and left for additional 2 h for the cross-linking reaction to reach optimal efficiency^62–64^. During the cross-linking reaction, a balance weight (200 g) is placed on the top of the mold to press against the PEG hydrogel. The fabricated molds were demolded from hydrogel after 2 h and the patterned hydrogel is ready for cell seeding.

### Cell culture and staining

The human colorectal cancer cell line (HCT-116) was obtained from American Type Culture Collection (ATCC) and used as tumor model cells in the work. The cell line was cultured in 5% CO_2_ at 37 °C in DMEM supplemented with 10% fetal calf serum (FBS; Invitrogen) and 1% antibiotic/antimycotic (AA, GIBCO). At 80% confluency, the HCT-116 cells were trypsinized into single-cell suspension and washed with a culture medium. 0.5 million cells were seeded in each of the 24 well plates with patterned PEG hydrogel. The cells were centrifuged at 300 g for 5 min or left to sediment by gravity. The cells form spheroid within 24-48 h and are stained with Calcein AM to indicate live cells.

The Human Lung fibroblasts (HLFs) and Human Umbilical Vein Endothelial Cells (HUVECs) were obtained from Lonza, using Fibroblast Growth Medium-2 (FGM-2) and Endothelial Cell Growth Basal Medium-2 (EBM-2), respectively. The cells were cultured under the same condition as HCT-116, and trypsinized with 0.25% Trypsin (R&D Systems) for 10 min, followed by 5 min, 300 g centrifuge. After discarding the supernatant, the collected cells for resuspended in the medium for the downstream experiments.

### Confocal imaging of microstructures

For the confocal scanning of the microstructures, the pre-polymer solution is mixed with 1% v/v Rhodamine B before UV polymerization. A Nikon Ti2 confocal microscope with NIS Elements 5.21.00 is used to scan the fluorescent-labeled microstructures.

### SEM imaging of microstructures

Samples are dehydrated in a 55 °C oven for 2 h before coating with 50 nm of chromium with E-beam evaporation at the Center for Photonics and Nanoelectronics (CPN) at Lehigh University. Then it is scanned with a tabletop Toshiba TM-1000 SEM.

### Reporting summary

Further information on research design is available in the Nature Portfolio Reporting Summary linked to this article.

## Supplementary information

Supplementary Information

Description of Additional Supplementary Files

Supplementary Movie 1

Supplementary Movie 2

Supplementary Movie 3

Supplementary Movie 4

Supplementary Movie 5

Supplementary Movie 6

Supplementary Movie 7

Reporting Summary

## Data Availability

The data generated in this study are provided in the Supplementary Information and the figshare database with the link 10.6084/m9.figshare.23575737 with CC BY4 license. All the other data are available from the corresponding authors upon request.
