# Supplementary material for: Rapid prototyping of high-resolution large format microfluidic device through maskless image guided in-situ photopolymerization
Source: Nat Commun. 2023 Jul 27;14:4520. doi: 10.1038/s41467-023-40119-x (PMC10374892; doi:10.1038/s41467-023-40119-x)
Supplement: Supplementary file 1 — Supplementary Information [file 41467_2023_40119_MOESM1_ESM.pdf]

Supplementary Informaiton For

Rapid Prototyping of High-resolution Large Format Microfluidic Device through Maskless Image Guided In-situ Photopolymerization

Ratul Paul[1], Yuwen Zhao[2], Declan Coster[3], Xiaochen Qin[2], Khayrul Islam[1,] and Yaling Liu[1,2, *]

[1] Department of Mechanical Engineering and Mechanics, Lehigh University, Bethlehem, PA 18015, USA
[2] Department of Bioengineering, Lehigh University, Bethlehem, PA 18015, USA
[3] Department of Electrical and Computer Engineering, Lehigh University, Bethlehem, PA 18015, USA

Supplementary Methods

Measurement of $d_{critical}$ from serial exposure

To determine $d_{critical}$, we first make 4 blank channels with the same thickness. 50% PEGDA 700 is used with the same UV dose for each row. The patterns of circular micropillars of the same diameter of 35 µm but different gap distances are projected in a single file as shown in Supplementary Supplementary Figure 1. After projecting all the patterns, they are imaged, and the diameter is measured using ImageJ. Micropillars of small gap distance <100 µm are found to have increased size over sequential projection due to accumulated curing in the same area. Micropillars with gap distance above 150 µm are found to remain the original target diameter, thus $d_{critical}$ is chosen as 150 µm for later array fabrication to avoid accumulated over-curing.

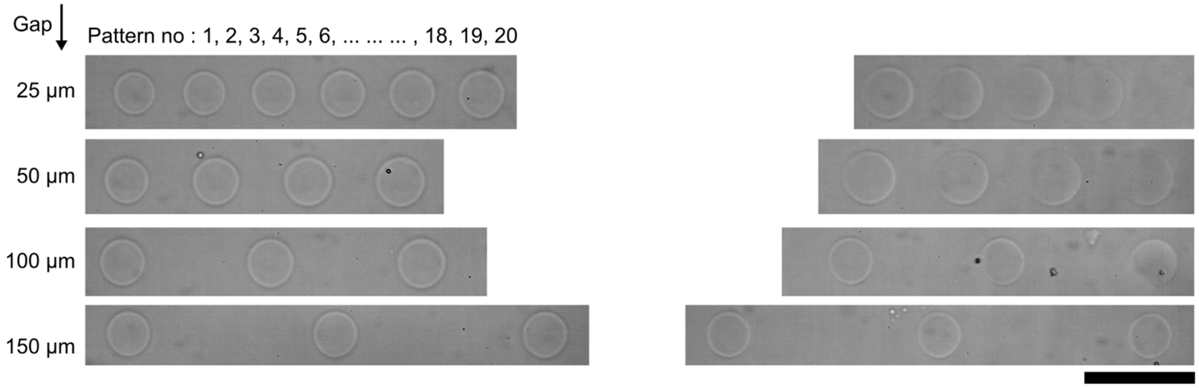

Supplementary Figure 1: The first and last few patterns in a serial exposure tests with different gap sizes. (Scale bar: 100 µm)

Feature printability and $d_{critical}$ characterization through experiment and simulation

The printability of a 35 µm feature in a ~20 µm tall channel with 50% PEGDA 700 is shown for different spacings. The experimental results are qualitatively compared to simulation using a simplified reaction-diffusion kinetic model [1] for a non-uniform and perfectly uniform illumination. The results are summarized in Supplementary Figure 2.

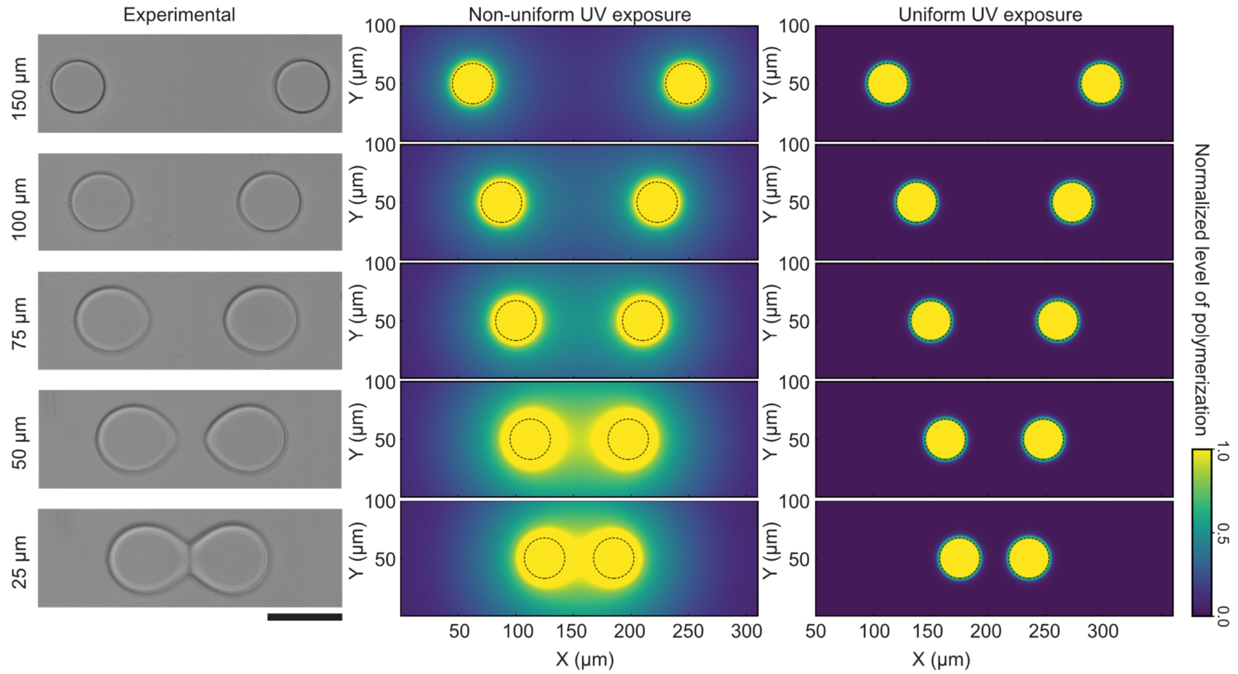

Supplementary Figure 2: Experimental and simulation results from projecting a pair of micropillars with 35 µm diameter at 25, 50, 75, 100 and 150 µm distance. The left column shows the result from the experiment. The middle and the right column show the results from the simulation for a non-uniform and perfectly uniform illumination, respectively (Scale bar: 50 µm)

Experimental setup

The experimental setup includes the microscope, UV projector, high-speed camera, and pressure pump and is centrally controlled by the computer with a Python code. The setup is shown in Supplementary Supplementary Figure 3**.**

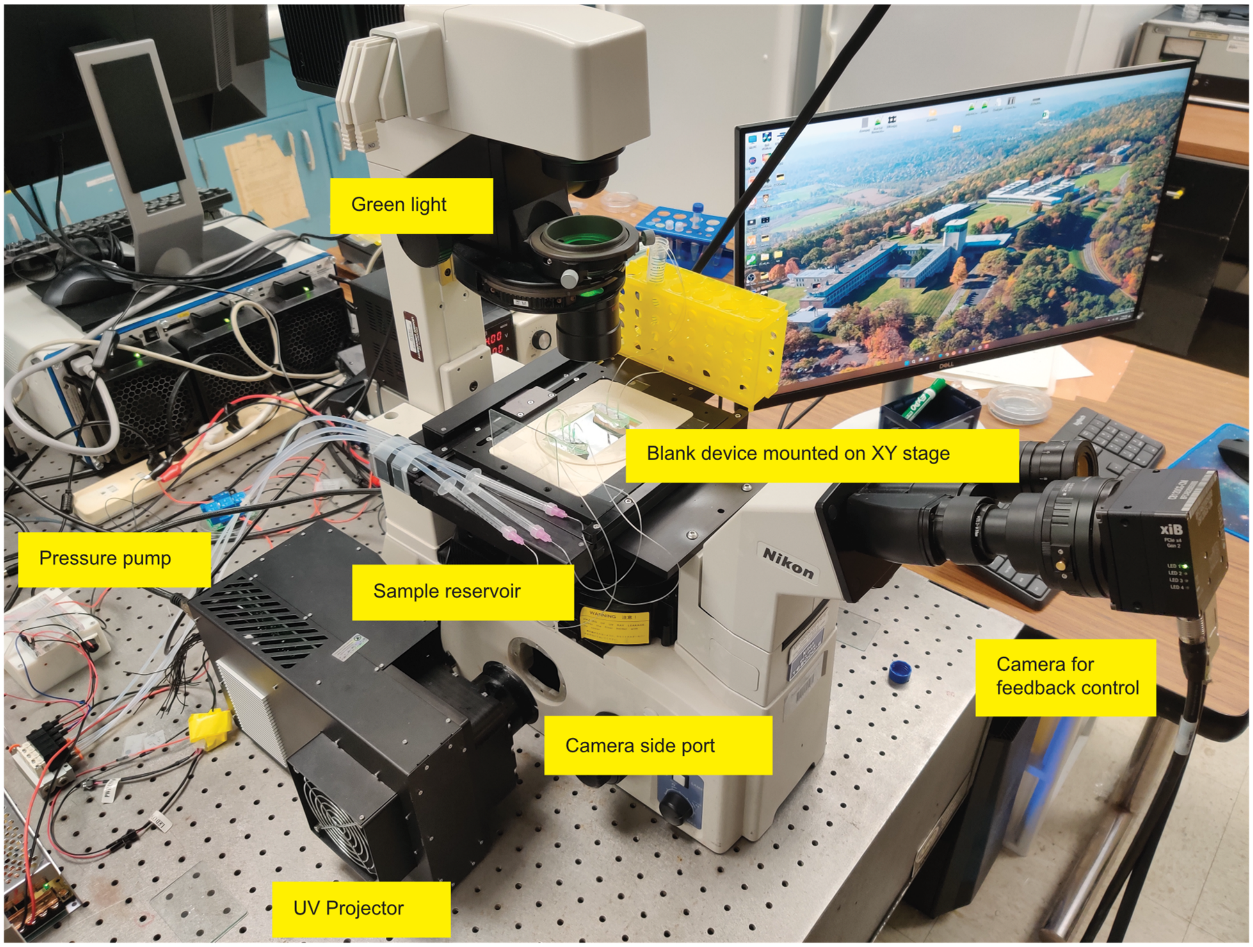

Supplementary Figure 3: Experimental setup of the Maskless Image Guided In-situ Photopolymerization Platform.

Blank channel fabrication

The fabrication of the blank device is described in the methods section. The blank device is shown in Supplementary Supplementary Figure 4. This specific type of blank device is made for DLD devices and thus has the sheath flow and two outlets. Configurations of inlet, outlet, and blank channels can be customized for other applications.

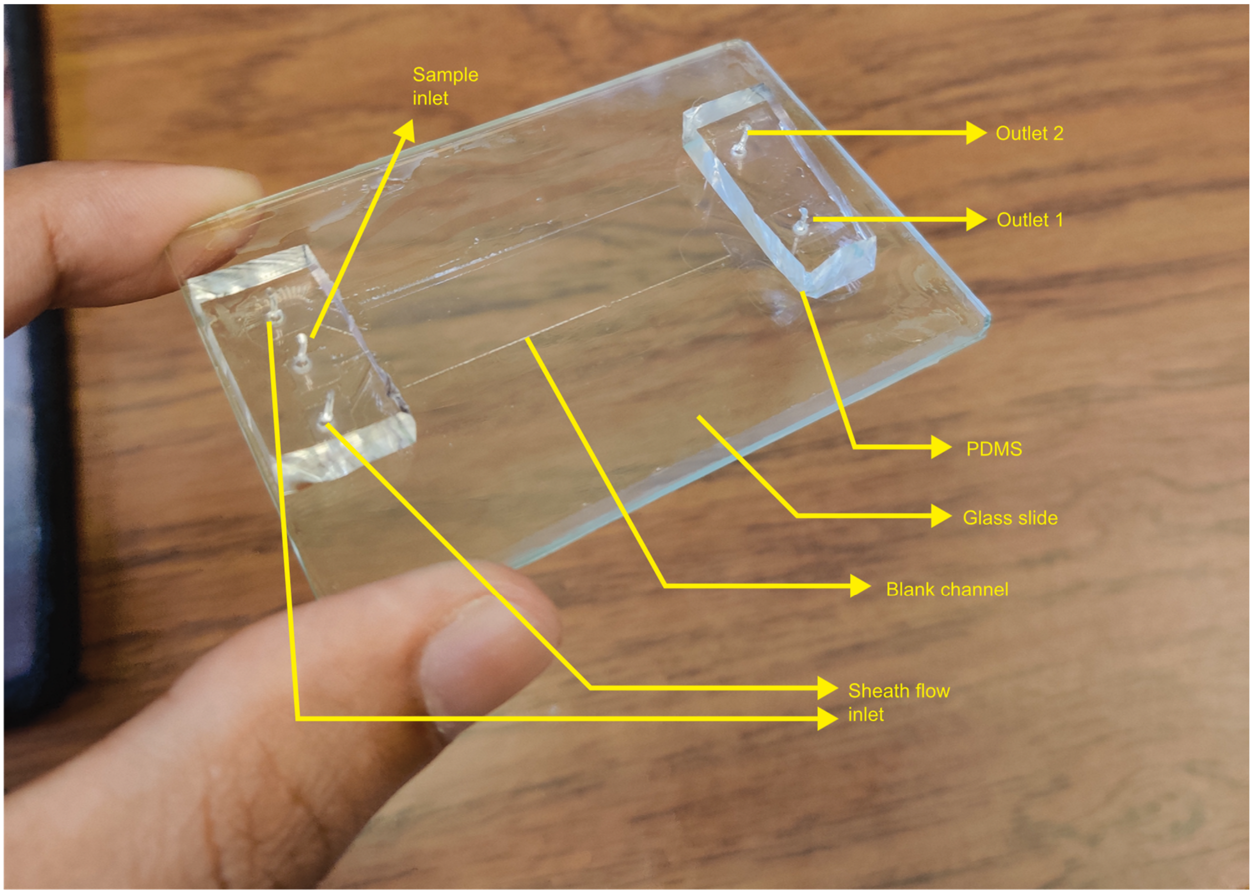

Supplementary Figure 4: A blank channel for DLD device fabrication

Supplementary Figures

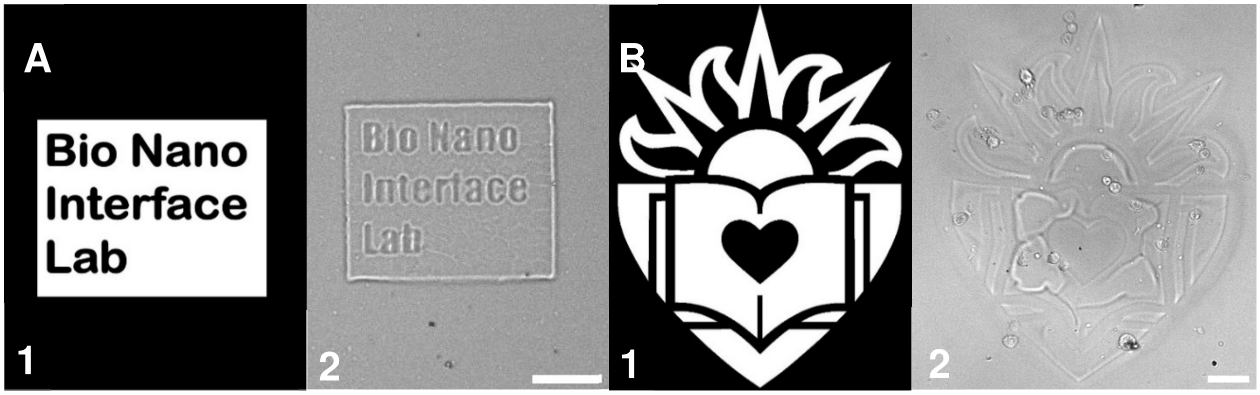

Supplementary Figure 5: Complex pattern printing by GelMA hydrogel. A) Logo of the Bionano interface Lab. B) Logo of Lehigh University. Figures 1 and 2 represent for digital mask and the GelMA geometry. Scale bar: 50 µm.

Supplementary References

1. Park, S. *et al.* Controlling uniformity of photopolymerized microscopic hydrogels. *Lab Chip* **14**, 1551–1563 (2014).
