# Supplementary material for: Rapid prototyping of high-resolution large format microfluidic device through maskless image guided in-situ photopolymerization
Source: Nat Commun. 2023 Jul 27;14:4520. doi: 10.1038/s41467-023-40119-x (PMC10374892; doi:10.1038/s41467-023-40119-x)
Supplement: Supplementary file 2 — Description of Additional Supplementary Files [file 41467_2023_40119_MOESM2_ESM.pdf]

Description of Additional Supplementary Files

File Name: Supplementary Movie 1
Description: Demonstration of edge tracking with the average greyscale value and a 10px wide digital mask, and dynamic projection triggering of individual patterns during curing. (Scale bar: 50 µm)

File Name: Supplementary Movie 2
Description: Simulation of the normalized polymer propagation in uniform and non-uniform illumination for 1 second exposure using the simplified free radical polymerization simulation.

File Name: Supplementary Movie 3
Description: Demonstration of image-based dynamic distortion correction by tracking critical points of a 'Star' shape and changing the projected pattern based on the deviation of the cured structure from the desired one. (a) Original movie of the curing process with the pattern being projected as overlay white color. (b) Edge tracking of the cured region. (c) A plot of the location of the five trenches from the center determined from the edge tracking along pre-defined lines. The location of the desired points is also shown in a vertical dashed line when the projection is stopped. (d) Shows a similar plot as (c) for the tips. This movie shows if the actual edges of the cured structures are trailing or leading to the desired location. (Scale bar: 100 µm)

File Name: Supplementary Movie 4
Description: Effect of UV power on the tracking of the patterns.

File Name: Supplementary Movie 5
Description: Movie of the experimental setup during the fabrication process.

File Name: Supplementary Movie 6
Description: The partial fabrication process of the DLD device shown in Figure 6(b) of the manuscript (selected part to illustrate the process).

File Name: Supplementary Movie 7
Description: The partial fabrication process of the device shown in Figure 7(b) of the manuscript (selected part to illustrate the process).
