# Supplementary material for: Rapid prototyping of high-resolution large format microfluidic device through maskless image guided in-situ photopolymerization
Source: Nat Commun. 2023 Jul 27;14:4520. doi: 10.1038/s41467-023-40119-x (PMC10374892; doi:10.1038/s41467-023-40119-x)
Supplement: Supplementary file 10 — Reporting Summary [file 41467_2023_40119_MOESM10_ESM.pdf]

nature portfolio

Corresponding author(s): Dr. Yaling Liu

Last updated by author(s): Jun 24, 2023

Reporting Summary

Nature Portfolio wishes to improve the reproducibility of the work that we publish. This form provides structure for consistency and transparency in reporting. For further information on Nature Portfolio policies, see our Editorial Policies and the Editorial Policy Checklist.

Statistics

For all statistical analyses, confirm that the following items are present in the figure legend, table legend, main text, or Methods section.

n/a	Confirmed	
☐	☒	The exact sample size (*n*) for each experimental group/condition, given as a discrete number and unit of measurement
☒	☐	A statement on whether measurements were taken from distinct samples or whether the same sample was measured repeatedly
☒	☐	The statistical test(s) used AND whether they are one- or two-sided<br>*Only common tests should be described solely by name; describe more complex techniques in the Methods section.*
☒	☐	A description of all covariates tested
☐	☒	A description of any assumptions or corrections, such as tests of normality and adjustment for multiple comparisons
☐	☒	A full description of the statistical parameters including central tendency (e.g. means) or other basic estimates (e.g. regression coefficient) AND variation (e.g. standard deviation) or associated estimates of uncertainty (e.g. confidence intervals)
☒	☐	For null hypothesis testing, the test statistic (e.g. *F*, *t*, *r*) with confidence intervals, effect sizes, degrees of freedom and *P* value noted<br>*Give P values as exact values whenever suitable.*
☒	☐	For Bayesian analysis, information on the choice of priors and Markov chain Monte Carlo settings
☒	☐	For hierarchical and complex designs, identification of the appropriate level for tests and full reporting of outcomes
☒	☐	Estimates of effect sizes (e.g. Cohen's *d*, Pearson's *r*), indicating how they were calculated

Our web collection on statistics for biologists contains articles on many of the points above.

Software and code

Policy information about availability of computer code

Data collection	Image collection was done in XIMEA CamTool and XIMEA API for Python v4.21.29, Confocal images were captured using Nikon NIS-Elements 5.21.00, Simulation data was collected using COMSOL 5.6.
Data analysis	Python 3.8 was used in Spyder 5.4.3 for the data, image and video processing, ImageJ 1.53n was used for brightfield, fluorescent and confocal image data processing.

For manuscripts utilizing custom algorithms or software that are central to the research but not yet described in published literature, software must be made available to editors and reviewers. We strongly encourage code deposition in a community repository (e.g. GitHub). See the Nature Portfolio guidelines for submitting code & software for further information.

Data

Policy information about availability of data

All manuscripts must include a data availability statement. This statement should provide the following information, where applicable:

- Accession codes, unique identifiers, or web links for publicly available datasets
- A description of any restrictions on data availability
- For clinical datasets or third party data, please ensure that the statement adheres to our policy

The data generated in this study are provided in the Supplementary Information and the figshare database with the link https://doi.org/10.6084/m9.figshare.23575737 with CC BY4 license. All the other data are available from the corresponding authors upon request.

Research involving human participants, their data, or biological material

Policy information about studies with human participants or human data. See also policy information about sex, gender (identity/presentation), and sexual orientation and race, ethnicity and racism.

Reporting on sex and gender	N/A
Reporting on race, ethnicity, or other socially relevant groupings	N/A
Population characteristics	N/A
Recruitment	N/A
Ethics oversight	N/A

Note that full information on the approval of the study protocol must also be provided in the manuscript.

Field-specific reporting

Please select the one below that is the best fit for your research. If you are not sure, read the appropriate sections before making your selection.

☒ Life sciences ☐ Behavioural & social sciences ☐ Ecological, evolutionary & environmental sciences

For a reference copy of the document with all sections, see nature.com/documents/nr-reporting-summary-flat.pdf

Life sciences study design

All studies must disclose on these points even when the disclosure is negative.

Sample size	For the statistical analysis, sample size was chosen based on the number of patterns occupying over 50% region of the devices.
Data exclusions	No data were excluded from the analysis
Replication	All fabrication methods reported in the manuscript were replicated at least 10 times after the methodology was established to ensure their reproducibility.
Randomization	When generating statistical data, the images were taken randomly from the devices whenever the full device was not included in the study.
Blinding	Blinding was not relevant to the study as most of the data were collected from images using image analysis software and when possible automated analysis was performed to minimize the risk of human error and bias.

Reporting for specific materials, systems and methods

We require information from authors about some types of materials, experimental systems and methods used in many studies. Here, indicate whether each material, system or method listed is relevant to your study. If you are not sure if a list item applies to your research, read the appropriate section before selecting a response.

Materials & experimental systems

n/a	Involved in the study	
☒	☐	Antibodies
☐	☒	Eukaryotic cell lines
☒	☐	Palaeontology and archaeology
☒	☐	Animals and other organisms
☒	☐	Clinical data
☒	☐	Dual use research of concern
☒	☐	Plants

Methods

n/a	Involved in the study	
☒	☐	ChIP-seq
☒	☐	Flow cytometry
☒	☐	MRI-based neuroimaging

Eukaryotic cell lines

Policy information about cell lines and Sex and Gender in Research

Cell line source(s)	HCT 116, PC3, Human Lung Fibroblast (hLF) : American Type Culture Collection (ATCC)<br>Peripheral Blood Mononuclear Cells (PBMC) : Penn Medicine (Human Immunology Core)<br>Human Umbilical Vein Endothelial Cells (HUVEC) : Lonza

Authentication	HCT 116, PC3, hLF cells are cultured cells and were authenticated by morphology. PBMCs were authenticated by morphology.
Mycoplasma contamination	Cell lines were not tested for mycoplasma contamination.
Commonly misidentified lines (See ICLAC register)	No commonly misidentified cell lines were used in the study.
